# The Changes in China's Forests: An Analysis Using the Forest Identity

**DOI:** 10.1371/journal.pone.0020778

**Published:** 2011-06-09

**Authors:** Lei Shi, Shuqing Zhao, Zhiyao Tang, Jingyun Fang

**Affiliations:** College of Urban and Environmental Sciences, and Key Laboratory for Earth Surface Processes of the Ministry of Education, Peking University, Beijing, China; Umea University, Sweden

## Abstract

Changes in forest carbon stocks are a determinant of the regional carbon budget. In the past several decades, China has experienced a pronounced increase in forest area and density. However, few comprehensive analyses have been conducted. In this study, we employed the Forest Identity concept to evaluate the changing status of China's forests over the past three decades, using national forest inventory data of five periods (1977–1981, 1984–1988, 1989–1993, 1994–1998, and 1999–2003). The results showed that forest area and growing stock density increased by 0.51% and 0.44% annually over the past three decades, while the conversion ratio of forest biomass to growing stock declined by 0.10% annually. These developments resulted in a net annual increase of 0.85% in forest carbon sequestration, which is equivalent to a net biomass carbon uptake of 43.8 Tg per year (1 Tg = 10^12^ g). This increase can be attributed to the national reforestation/afforestation programs, environmentally enhanced forest growth and economic development as indicated by the average gross domestic product.

## Introduction

Forests cover four billion hectares (31%) of the Earth's landmass [1] and contain over 75% of all carbon in vegetation [2]. They can provide renewable raw materials and natural amenities, protect land and water resources, harbor biological diversity and mitigate climate change [3], [4]. Forest area, growing stock, biomass, and sequestrated carbon are valuable indicators that embody these functions. Area is the first indicator of the relative importance of forests in a country or region, and estimates of changes in forest area over time and space can characterize deforestation and reforestation/afforestation. An index of growing stock can provide information on existing wood resources, and its estimates constitute the basis for estimation of carbon dioxide (CO_2_) sequestered by forest biomass. A good understanding of the carbon dynamics of forests is crucial for climate change mitigation. Therefore, evaluating forest attributes is of great significance in the development of macro-policy and environmental monitoring for a country or region. For this reason, many studies have focused on forest resource assessments [5]–[15]. However, most of these assessments have only analyzed one or two forest attributes (i.e., forest expanse, growing stock, biomass, or carbon stock). Such assessments do not offer an integrated understanding of the state of dynamic and multifaceted forests.

For any forest, carbon stock (*Q*) can be calculated from the following four measurable variables: forest area (*A*), forest growing stock density (*D*), the conversion ratio of forest biomass to growing stock (*B*, *cited below as the* “*conversion ratio*”) and carbon concentration (*C*), so that *Q* = *A*×*D*×*B*×*C*. Changes in any of these four components can cause changes in forest carbon stocks. To better understand the relative contribution of each attribute, it is necessary to separate the forest carbon stocks into different components. To do so, we need a multivariate model to decompose the effects of changes in different components on the carbon sequestration of forests. The Forest Identity method, developed by Kauppi et al. [16] and Waggoner [17], provides an efficient approach to such an analysis. It is a conceptual framework devised to define these valued attributes and integrate them quantitatively with logical weights, and it can therefore be used to obtain a comprehensive assessment of the forest resources of a country or a region.

**Figure 1:**
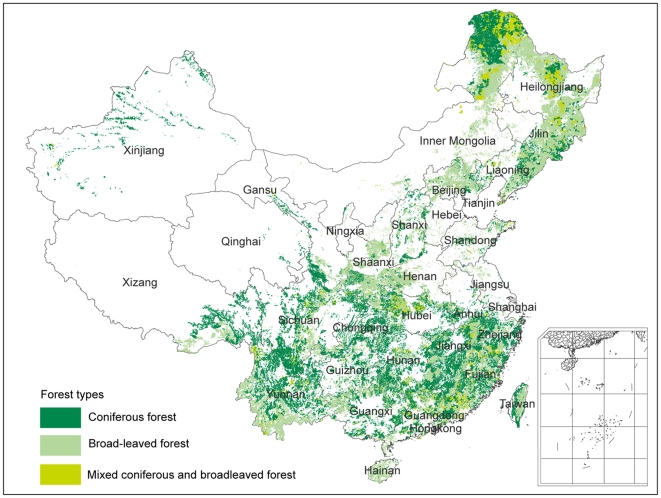
Geographical distribution of forests in China based on the data of the sixth forest inventory (1999–2003). Forests are grouped into three types: coniferous forest, broadleaved forest, and coniferous and broadleaved mixed forest. The background map shows the administrative divisions of China.

China has experienced a large-scale practice of reforestation and afforestation over the past several decades [18], [19]. According to recent information, forests cover 195.4 million ha of the country [20]. The types of forests range from tropical to boreal (Fig. 1). Therefore, a comprehensive assessment of China's forest resources is important for clarifying the nature of regional and global forest change.

In this study, we used the Forest Identity concept and national forest inventory data of five time periods (1977–1981, 1984–1988, 1989–1993, 1994–1998, and 1999–2003) to evaluate the status and change of China's forests over the past three decades, at both the provincial and the national scales.

## Results

### Status and change of China's forests at the national scale

Using inventory data and the continuous biomass expansion factor approach developed by Fang et al. [5], [22], we calculated the area (*A*), the growing stock density (*D*) and the conversion ratio (*B*) for China's forests. Using a constant carbon concentration (*C*, the ratio of carbon content to total biomass) of 0.5 Mg C/Mg, we then estimated the biomass carbon stock of China's forests for each period using Eq. (3) (see Materials and Methods). As shown in Table 1 (Part I), China's forest biomass carbon stock increased from 4.70 to 5.86 Pg C (1 Pg = 10^15^ g) over the study period.

**Table 1:** Area (*A*), growing stock density (*D*), the conversion ratio of biomass to growing stock (*B*), and biomass carbon stock (*Q*) (Part I), and the relative annual rates of change (Part II) of these attributes for China's forests from 1977 to 2003 at the national level.

Part I	Time span	*A* (10^4^ ha)	*D* (m^3^/ha)	*B* (Mg/m^3^)	*Q* (Pg C)
	1977–1981	12300.2	77.29	0.988	4.70
	1984–1988	13127.2	73.38	1.010	4.86
	1989–1993	13926.6	76.87	0.997	5.33
	1994–1998	12919.9	78.06	0.996	5.02
	1999–2003	14280.3	84.73	0.969	5.86
Part II	**Time span**	***a*** ** (%)**	***d*** ** (%)**	***b*** ** (%)**	***q*** ** (%)**
	1977–2003	0.51	0.44	−0.10	0.85

From Eqs. (7) and (8), we estimated the annual rates of change of these forest attributes over the study period. The estimated rates for forest area (*a*), growing stock density (*d*), the conversion ratio (*b*) and constant carbon concentration (*c*) were 0.51%, 0.44%, −0.10%, and 0, respectively. Based on Eq. (4), we calculated a net biomass carbon increase of 0.85% ( = 0.51%+0.44%−0.10%+0) annually in China's forests (Part II in Table 1), which is equivalent to a carbon sequestration of 43.8 Tg per year.

### Changes in China's forests at the provincial scale

#### Change in forest growing stock (*v*)

**Figure 2:**
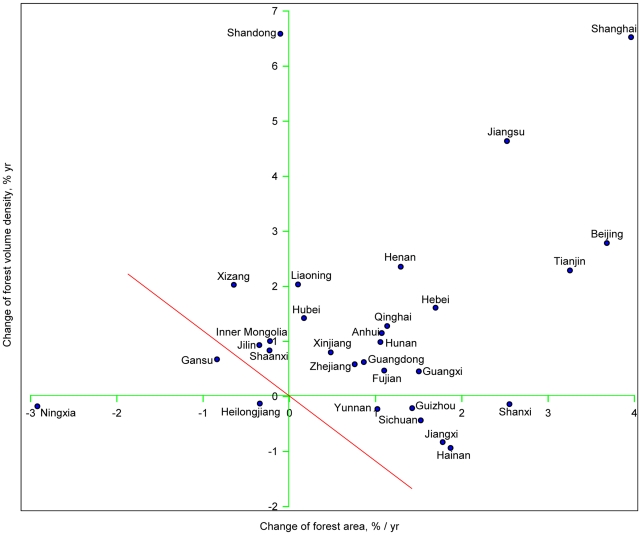
A synoptic chart showing the changes in China's forests over the past three decades. On the chart, the horizontal axis is the relative annual change of forest area (*a*), and the vertical axis is the relative annual change of forest volume density (*d*). The growing stock (*v*) was increasing in the provinces above the diagonal line *a = −d.*

**Figure 3:**
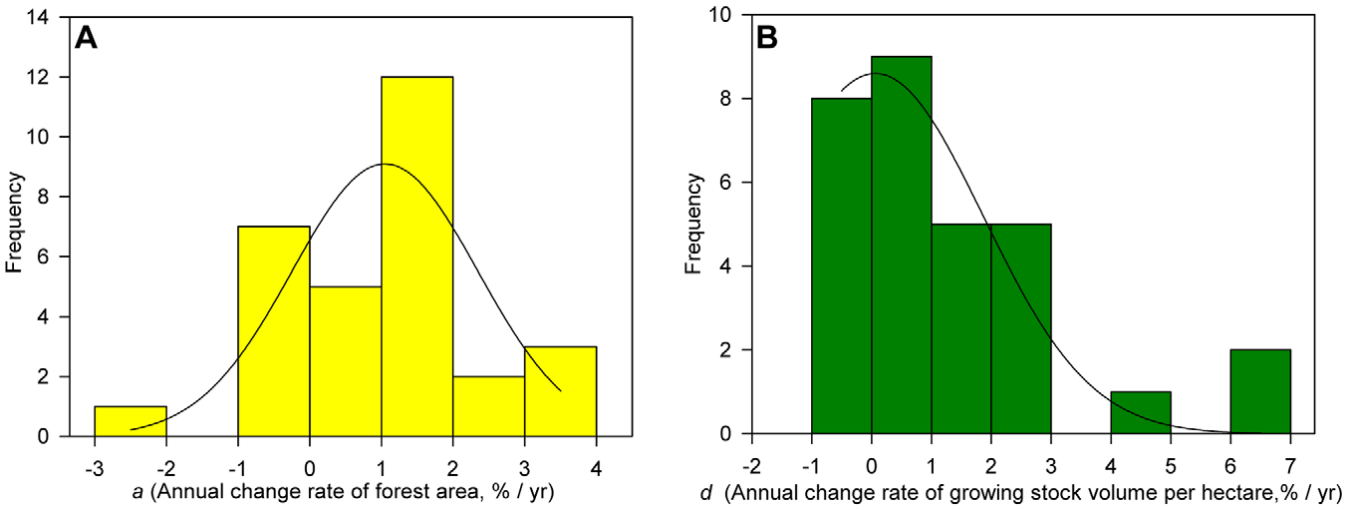
Frequency maps of annual rates of change in area (*a*) (A) and volume density (*d*) (B) for China's forests over the past three decades.

As shown in Eq. (5), the change in forest growing stock is the sum of changes in both area and growing stock density. Forest area increased over the study period in most provinces except Ningxia (−2.9%), Gansu (−0.84%), Xizang (−0.64%), Jilin (−0.35%), Heilongjiang (−0.34%), Shaanxi (−0.23%), Inner Mongolia (−0.22%) and Shandong (−0.10%) (Fig. 2). Of the 22 provinces with increasing forest area, five increased by <1.0%, 12 increased by 1.0∼2.0%, and five increased by >2.0% annually (Fig. 3A). These results suggest that afforestation or reforestation has occurred in 73.3% (22 out of 30) of China's provinces and that 16.7% (5 out of 30) experienced rapid forest expansion (an annual increase of >2.0%) over the study period.

Forest density has increased in most provinces except Hainan (−0.94%), Jiangxi (−0.83%), Sichuan (−0.44%), Yunnan (−0.23%), Guizhou (−0.21%), Ningxia (−0.18%), Shanxi (−0.14%) and Heilongjiang (−0.13%) (Fig. 2). Of the provinces with increasing density, nine showed increases at an annual rate of <1.0%, five at 1.0∼2.0%, and eight at >2.0% (Fig. 3B).

A synoptic view (Fig. 2) of the observed variations in forest area and density revealed the direction (i.e., change for the worse or change for the better) and rate of change of the forest growing stock in each province over the study period. The change in growing stock (*v*) showed an increase for the provinces above the diagonal line (*a* = −*d*, red line in Fig. 2) and a decrease in the remaining provinces. Overall, the forest growing stock increased in 27 provinces, of which 16 showed increases in both area and density, six showed increases in forest area but decreases in density (i.e., Yunnan, Hainan, Jiangxi, Sichuan, Guizhou and Shanxi), and five increased in density but decreased in forest area (Jilin, Shaanxi, Inner Mongolia, Xizang and Shandong). The three provinces of Ningxia, Heilongjiang and Gansu showed a decline in growing stock. A decrease in both area and density resulted in the decrease of growing stock in Ningxia and Heilongjiang, whereas a smaller increase in density combined with a larger decrease in area led to a net decline of growing stock in Gansu.

#### Change in biomass (*m*) or carbon sequestration (*q*)

As indicated in Eq. (6), change in biomass (*m*) can result from change in area and density (i.e., *a* and *d*), but it can also result from change in the conversion ratio (*b*). Because we used a constant carbon concentration (C = 0.5 Mg C/Mg, or *c* = 0), the change in biomass (*m*) is equal to the change in carbon sequestration (*q*).

**Figure 4:**
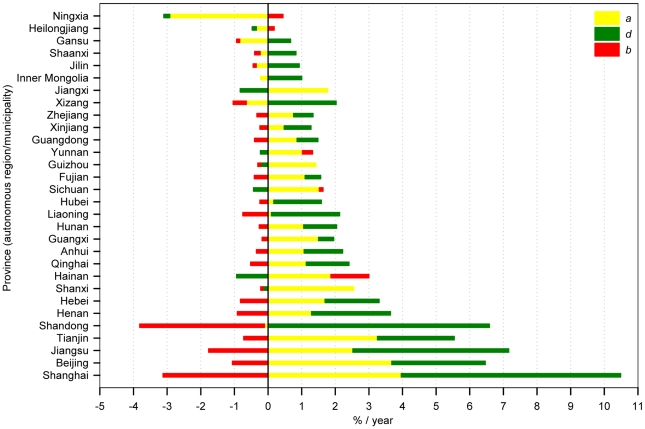
Rates of change of forest area (*a*), growing stock density (*d*), and the conversion ratio of biomass to growing stock (*b*) in each province over the past three decades.

Figure 4 illustrates the contributions of these three attributes (i.e., *a*, *d* and *b*) to the change in biomass or carbon sequestration. Generally, the attributes *d* and *b* had opposite effects, and the rate of change in *m* or *q* was slower than that in growing stock, although *m* or *q* and *v* changed in the same direction. Specifically, the forests in 27 provinces functioned as a carbon sink and those in the remaining three provinces (Ningxia, Heilongjiang and Gansu) as a carbon source over the study period.

**Figure 5:**
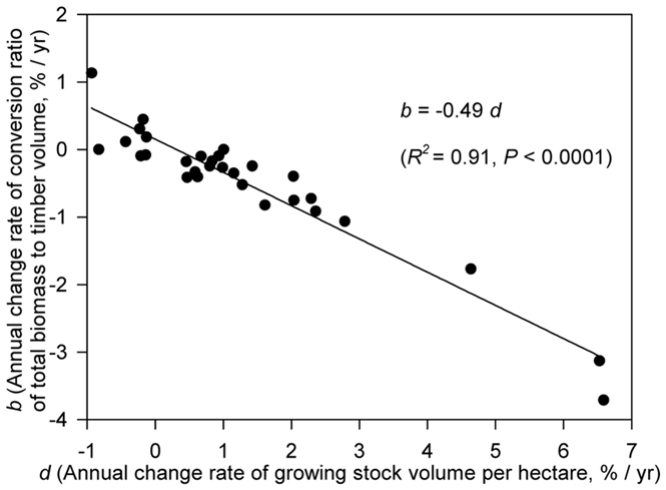
Relationship between relative annual changes in the conversion ratio of biomass to growing stock (*b*) and in the growing stock density (*d*).

We further examined the relationship between relative annual changes in growing density (*d*) and the conversion ratio (*b*), and found a good linear correlation between these two attributes (*b* = −0.45*d*, *R* ^2^  = 0.90) (Fig. 5). The results showed that *b* is strongly dependent on *d* and thus suggested that the conversion ratio is province-dependent, a result consistent with previous findings that the conversion ratio varies with stand age, site class and stand density [5], [21], [28]–[29], [32]–[33].

## Discussion

Over the past three decades, forest area and growing stock density in China increased at the annual rates of 0.51% and 0.44%, respectively. As a result, 43.8 Tg carbon was sequestered annually by China's forests. Overall forest expansion and growth over the past several decades and the resulting carbon uptake by China's forests have also been observed in previous studies [5], [22], [34]. These increases are primarily attributed to several national reforestation and afforestation programs implemented since the 1980s (such as the River Protection Forest Project, the Natural Forest Protection Program and the Conversion of Cropland to Forest Program) [18], [19], [35]. A lengthening of the growing season induced by warming climate and increasing summer precipitation in China could also have contributed to this growth [36]–[38]. Although several studies have reported that elevated CO_2_ and natural nitrogen deposition are factors enhancing forest growth [39]–[40], no such evidence has been observed for China's forests.

Despite an overall increase in both area and density for China's forests, declines in forest area and/or growth occurred in some regions. For example, eight provinces (Ningxia, Gansu, Xizang, Jilin, Heilongjiang, Shaanxi, Inner Mongolia and Shandong) experienced a forest shrinkage, and eight provinces (Hainan, Jiangxi, Sichuan, Yunnan, Guizhou, Ningxia, Shanxi and Heilongjiang) underwent a decline in forest density (Figs. 2 and 4). Evidently, most of the provinces with shrinking forest are located in the arid region. Those with declining forest density are mainly in the Southwest China, where the average forest stock density is relatively high. Taken together, these findings suggest that climate and the degradation of old forests might have contributed to the reduction of forest area/density in those regions. In addition, logging and wildfire are important perturbation factors that have caused the decline in both forest area and density in the Northeast China [37], [41].

**Figure 6:**
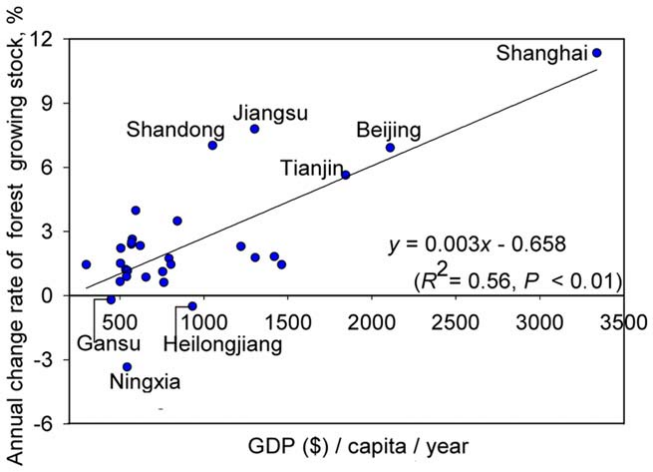
The relative annual rate of change of forest growing stock (*a + d*) in provinces plotted as a function of their average GDP (y = 0.003x−0.658). The change in forest growing stock was measured over the period 1977–2003. The GDP values (U.S. dollars) were for 1999.

It is generally recognized that environmental degradation (including deforestation) and economic development (human activities) are closely related, a pattern described by an Environmental Kuznets Curve (EKC) [42]–[45]. The EKC implies that in poor areas (with a low average income), economic development leads to ecological deterioration, whereas in rich areas (with a relatively high average income), the awareness of environmental protection increases. In the rich areas, economic development does not inflict environmental damage; instead, it promotes the sound development of environment. However, our research shows that the relative annual rate of change of growing stock and the average GDP (GDP per capita) in 1999 showed a significant positive relationship (*R* ^2^ = 0.56, *P*<0.01) (Fig. 6), which does not support the EKC.

A comparison of Fig. 2 with Fig. 6 shows that the provinces with higher increases in forest area and/or density and a resulting faster increase in growing stock were those having a high average GDP, such as Shanghai, Beijing, Tianjin, Jiangsu and Shandong. In contrast, the average GDP was relatively low in the provinces with declining forest growing stock, such as Ningxia and Gansu. The exception to this pattern is Heilongjiang, where decreases in both area and density were observed despite the province's relatively high average GDP. The degradation of forests in Heilongjiang Province was mainly attributed to overharvest and wildfire [37], [41].

Apparently, economic development (average GDP) in a region or country affects its environment greatly. Notably, coevolution between economic development and environmental protection is commonly recognized in East Asian countries. Promotion of the environment first appeared in Japan (in ∼1950s), then in South Korea (1960–1970s), and subsequently in China (1980s) and may be expected in North Korea and Mongolia in the near future [46].

## Materials and Methods

### National Forest Inventory (NFI)

The National Forest Inventory (NFI) program in China began in the 1970s. Seven inventories have been taken (i.e., 1973–1976, 1977–1981, 1984–1988, 1989–1993, 1994–1998, 1999–2003 and 2004–2008) [20]. These inventories were well designed and statistically sound. Over this period, a total of 415,000 permanent and temporary plots have been set up across the forested areas of the country. Systematic sampling with a grid of 2 km ×2 km or 4 km ×4 km has been used, depending on the forest region. In each grid, at least one plot with an area of 10 m ×10 m was investigated. Except during the first inventory (1973–1976), growing stock (by age class and by forest type) and forest area have been documented at the provincial level. In this study, we used the inventories for 1977–1981, 1984–1988, 1989–1993, 1994–1998, and 1999–2003. The data from the most recent inventory (2004–2008) were not yet available. The first inventory reported overall provincial-level information but did not stratify the data by forest type.

Unfortunately, these forest inventories provide only information of commercial significance (growing stock). They do not include detailed information about forest biomass. Using inventory data and the continuous Biomass Expansion Factor (BEF) method, Fang et al. [5], [21] estimated the biomass (including the stem, branch, root, and leaf biomass values for all living trees and shrubs) of each forest type and total biomass at a provincial level. In this paper, these estimates were used to evaluate the changes in China's forest biomass and the conversion ratio of forest biomass to growing stock for each province (except Taiwan, Hongkong, and Macao).

Notably, the tree canopy cover threshold defining a forest was changed from the value of 30% used in the first four inventories to a value of 20% for the fifth NFI (1994–1998) and subsequent inventories. To make the information on forest area and growing stock before and after the fifth NFI comparable, we used a linear model developed by Fang et al. [22] to adjust forest area and growing stock data reported before the fifth NFI.

### Methods

#### Forest Identity

For any forest, expanse (area), growing stock, biomass and carbon can be linked using Eqs 1–3. The Forest Identity method defines these four valued attributes by using measurable variables, and it quantitatively and logically integrates their changes into a causal relationship (i.e., Eqs 4–6) [16]–[17].



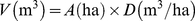
(1)

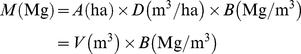
(2)

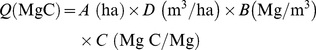
(3)

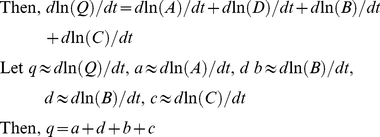
(4)

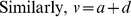
(5)

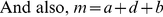
(6)


where *V*, *M*, *Q, A*, *D*, *B* and *C* represent growing stock (m^3^), biomass (Mg), forest carbon stock (Mg C), area (ha), growing stock density (m^3^/ha), the conversion ratio of biomass to growing stock (Mg/m^3^) and carbon concentration in biomass (Mg C/Mg) at the provincial or national level, respectively; *v*, *m*, *q*, *a*, *d*, *b* and *c* represent the corresponding derivatives of these attributes with respect to time.

Notably, although the conversion ratio was used as a constant in the earlier studies [23], [24], recent studies have shown that it varies with stand age, site class and stand density and that applying a constant conversion ratio generally underestimates biomass in young stands and overestimates biomass in old stands [5], [25]–[28]. Further studies indicate that the conversion ratio varies with growing stock density at a provincial and national level [26], [29] and thus suggest a state-dependent conversion ratio. Moreover, the carbon concentration in biomass (*C*) is commonly treated as a constant ratio of ∼50% (usually varying from 48% to 53%) of carbon content in dry mass of forest [24], [30]–[31], and this ratio is also employed in this study.

#### Annual change rate in forest attributes

We used Eqs. (7) and (8) to obtain the derivatives of the forest attributes with respect to time:
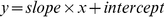
(7)


where *y* represents the forest attributes (i.e., area, growing stock density, the conversion ratio, or carbon content) at the provincial or national level, *slope* denotes the amplitude and direction of annual absolute change for each forest attribute, and *x* represents the corresponding periods of NFI. The years used here to represent the NFI periods were the medians for each time period: 1979 (1977–1981), 1986 (1984–1988), 1991 (1989–1993), 1996 (1994–1998), and 2001 (1999–2003), respectively.

Therefore, the relative annual change rate (*RR*, %/yr) of the forest attributes can be expressed as follows:
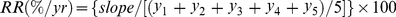
(8)


where *slope* is the regression coefficient in Eq. (7), and *y*_1_, *y*_2_, *y*_3_, *y*_4_ and *y*_5_ denote the corresponding forest attributes for the inventories of 1977–1981, 1984–1988, 1989–1993, 1994–1998, and 1999–2003, respectively. In other words, the relative annual change rate (*RR*, %) defined here is equivalent to *q*, *a*, *d* or *b* mentioned above.
